# Reducing NF-κB Signaling Nutritionally is Associated with Expedited Recovery of Skeletal Muscle Function After Damage

**DOI:** 10.1210/clinem/dgab106

**Published:** 2021-02-26

**Authors:** Tom S O Jameson, George F Pavis, Marlou L Dirks, Benjamin P Lee, Doaa R Abdelrahman, Andrew J Murton, Craig Porter, Nima Alamdari, Catherine R Mikus, Benjamin T Wall, Francis B Stephens

**Affiliations:** 1 Nutritional Physiology Group, Department of Sport and Health Sciences, College of Life and Environmental Sciences, University of Exeter, Exeter, Devon EX1 2LU, UK; 2 Institute of Biomedical and Clinical Sciences, University of Exeter Medical School, University of Exeter, Exeter, Devon EX1 2LU, UK; 3 Department of Surgery, University of Texas Medical Branch, Galveston, TX 77555, USA; 4 Beachbody LLC, Santa Monica, CA 90404, USA

**Keywords:** inflammation, muscle damage, deuterated water, protein-polyphenol

## Abstract

**Context:**

The early events regulating the remodeling program following skeletal muscle damage are poorly understood.

**Objective:**

The objective of this study was to determine the association between myofibrillar protein synthesis (myoPS) and nuclear factor-kappa B (NF-κB) signaling by nutritionally accelerating the recovery of muscle function following damage.

**Design, Setting, Participants, and Interventions:**

Healthy males and females consumed daily postexercise and prebed protein-polyphenol (PP; *n* = 9; 4 females) or isocaloric maltodextrin placebo (PLA; *n* = 9; 3 females) drinks (parallel design) 6 days before and 3 days after 300 unilateral eccentric contractions of the quadriceps during complete dietary control.

**Main Outcome Measures:**

Muscle function was assessed daily, and skeletal muscle biopsies were taken after 24, 27, and 36 hours for measurements of myoPS rates using deuterated water, and gene ontology and NF-κB signaling analysis using a quantitative reverse transcription PCR (RT-qPCR) gene array.

**Results:**

Eccentric contractions impaired muscle function for 48 hours in PLA intervention, but just for 24 hours in PP intervention (*P* = 0.047). Eccentric quadricep contractions increased myoPS compared with the control leg during postexercise (24–27 hours; 0.14 ± 0.01 vs 0.11 ± 0.01%·h^-1^, respectively; *P* = 0.075) and overnight periods (27–36 hours; 0.10 ± 0.01 vs 0.07 ± 0.01%·h^-1^, respectively; *P* = 0.020), but was not further increased by PP drinks (*P* > 0.05). Protein-polyphenol drinks decreased postexercise and overnight muscle *IL1R1* (PLA = 2.8 ± 0.4, PP = 1.1 ± 0.4 and PLA = 1.9 ± 0.4, PP = 0.3 ± 0.4 log_2_ fold-change, respectively) and *IL1RL1* (PLA = 4.9 ± 0.7, PP = 1.6 ± 0.8 and PLA = 3.7 ± 0.6, PP = 0.7 ± 0.7 log_2_ fold-change, respectively) messenger RNA expression (*P* < 0.05) and downstream NF-κB signaling compared with PLA.

**Conclusion:**

Protein-polyphenol drink ingestion likely accelerates recovery of muscle function by attenuating inflammatory NF-κB transcriptional signaling, possibly to reduce aberrant tissue degradation rather than increase myoPS rates.

## Introduction

Skeletal muscle is a highly plastic tissue with the ability to remodel myofibrillar structures following damage. Voluntary eccentric muscle contractions can induce substantial and immediate ultrastructural damage to myofibrillar structures (1). This manifests functionally as a transient impairment in force-generating capacity, with the greatest detriment occurring within 48 hours (2). To restore myofibrillar structure and function, a remodeling program is initiated involving the simultaneous and rapid activation of inflammatory (3), myogenic (4), transcriptional (5–8), translational (9–12), and post-translational (11, 13–15) processes. However, intervention studies that simultaneously investigate multiple remodeling processes are limited, particularly in humans. Thus, our understanding of how this remodeling program regulates the recovery of muscle function following myofibrillar damage remains incomplete.

Myofibrillar damage increases the demand for the synthesis of replacement myofibrillar protein (9). Recently, we demonstrated that the damage-induced impairment in muscle function occurs in parallel with a marked increase in myofibrillar protein synthesis (myoPS) rates and mammalian target of rapamycin (mTOR) phosphorylation, but only during overnight sleep between 24 and 48 hours following damaging eccentric contractions (ECs) (16). We then demonstrated that, relative to a carbohydrate placebo (PLA), postexercise whey protein ingestion potentiated overnight myoPS rates after ECs, which was associated with a profound improvement in muscle function. It has been shown that casein protein ingested before sleep potentiates overnight myoPS rates following traditional resistance-type exercise (17, 18). Consequently, if overnight myoPS rates are limiting to the recovery of muscle function after ECs, then presleep, in addition to postexercise protein ingestion, would be expected to further accelerate the recovery of muscle function following ECs.

Activation of the inflammatory nuclear factor-kappa B (NF-κB) transcription factor coordinates the immune and cell cycle responses to myofibrillar damage and is known to promote protein breakdown and inhibit protein synthesis (19). NF-κB DNA binding occurs rapidly (3 hours) in response to damaging ECs, in parallel with upregulated muscle gene expression upstream and downstream of NF-κB (8). Moreover, the increase in NF-kB phosphorylation 48 hours following ECs parallels the greatest impairment to muscle function (14, 15). Interestingly, whey protein (14), antioxidants (15), and polyphenols (20, 21) are known to both attenuate NF-κB activation and accelerate recovery of muscle function following damage. However, to our knowledge, the temporal association between NF-κB transcriptional signaling, myoPS, and the recovery of muscle function from damage has not yet been directly investigated.

Presently, we hypothesized that overnight myoPS rates and elevated inflammatory NF-κB signaling may be limiting the recovery of muscle function after damaging ECs. Consequently, we hypothesized that targeting these pathways with a postexercise and prebed protein-polyphenol (PP) intervention would accelerate the recovery of muscle function by increasing overnight myoPS rates and suppressing inflammatory NF-κB signaling.

## Materials and Methods

### Participants

Eighteen young, healthy males (*n* = 11) and females (*n* = 7) (age: 23 ± 1 years, body mass index [BMI]: 23.9 ± 1 kg·m^-2^) volunteered to take part in the present study. The participants’ characteristics are displayed in Table 1. Participants were recreationally active, defined as participating in sporting activities > 2 hours per week, but had not engaged in structured exercise training in the 6 months preceding participation. Female participants not using an oral contraceptive were at day 6 to 12 of a regular menstrual cycle (ie, midfollicular phase) during days 7 to 9 of the study protocol. Participants were deemed healthy based on their response to a routine medical screening questionnaire (ie, absence of any musculoskeletal injury, medication use, diagnosed metabolic or cardiovascular impairment, or motor disorders). Individuals routinely consuming nutritional supplements and nonsteroidal anti-inflammatory drugs or had a habitual protein intake of <0.8 g·kgBM^-1^·d^-1^ were excluded from participation. Participants were informed of the experimental procedures, potential risks, and the purpose of the study before providing full written consent. The study was approved by the Sport and Health Sciences Ethics Committee of the University of Exeter (REF NO. 161026/B/06) in accordance with standards for human research as outlined in the declaration of Helsinki. The study is part of a larger investigation registered at ClinicalTrials.Gov (ID: NCT02980900).

**Table 1.** Participant characteristics

	PLA	PP
	(*n* = 9)	(*n* = 9)
Sex	M = 6, F = 3	M = 5, F = 4
Age (y)	22 ± 1	23 ± 2
Body mass (kg)	78 ± 4	69 ± 4
Height (cm)	176 ± 1	173 ± 3
BMI (kg·m^-2^)	25 ± 1	23 ± 1
Total eccentric work (J·kg^-1^)	642 ± 43	574 ± 44
Habitual protein (g·kgBM^-1^·day^-1^)	1.1 ± 0.1	1.4 ± 0.2
Controlled protein (excluding condition) (g·kgBM^-1^·day^-1^)	1.2 ± 0.1	1.2 ± 0.1
Controlled protein (including condition) (g·kgBM^-1^·day^-1^)	1.2 ± 0.1	1.8 ± 0.1^a^

Values represent mean ± SEM. Total eccentric work represents the total volume of eccentric work performed during the unilateral eccentric exercise protocol on day 7 of the experimental period normalized for body mass. Habitual protein represents participants 3-day average habitual protein intake obtained from a food diary. Controlled protein represents the 14-day average protein intake provided by the dietary control. Comparisons between PLA and PP were performed with separate unpaired t-tests.

Abbreviations: BMI, body mass index; F = female; M = male; NS, nonsignificant (*P* > 0.05); PLA, placebo; PP, protein-polyphenol; y, years.

^a^Difference in controlled protein intake (including condition) between PLA and PP.

### Familiarization

Following screening and acceptance onto the study, all participants were familiarized with the exercise equipment and protocols at least 48 hours prior to the initiation of the experimental period. All exercise was performed on a Biodex System 3 isokinetic dynamometer (Biodex Medical Systems, Shirley, New York, USA). Participants were seated with 85^o^ of hip flexion and extraneous movement was restrained using shoulder, hip, and thigh straps. The Biodex configuration was recorded during the familiarization session and remained the same for each participant during sequential tests.

### Experimental protocol

**Figure 1.**
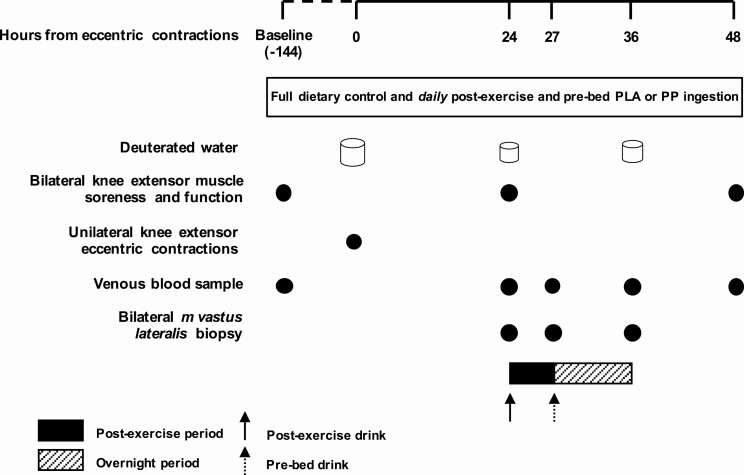
Schematic representation of the experimental protocol and the location of the postexercise and overnight periods. Participants were provided with full dietary control (1.2 g·kgBM^-1^·d^-1^ dietary protein) and postexercise and prebed protein-polyphenol (*n* = 9) or isocaloric maltodextrin placebo (*n* = 9) drinks were consumed daily. Loading of orally consumed 70% deuterated water began at ~8:00 am on day 7, with 8 x 6 ml·kg^-1^ doses consumed every 1.5 hours; body water enrichment was maintained thereafter with daily doses of 0.54 ml·kg^-1^. Unilateral eccentric knee extensor contractions were performed at ~7:00 pm on day 7 (*t* = 0 hours), and muscle soreness and function were measured at baseline and after 24 and 48 hours. Bilateral muscle biopsies were obtained 24, 27, and 36 hours following eccentric contractions, and venous blood samples were obtained at baseline and 27, 27, 36, and 48 hours following eccentric contractions. Abbreviations: PLA, placebo; PP, protein-polyphenol.

An overview of the experimental protocol is shown in Fig. 1. Participants were randomly assigned to 1 of 2 parallel, fully controlled nutritional intervention groups and completed a 9-day study period. Nutritional conditions consisted of either daily postexercise and prebed PP or PLA drinks (see “Experimental Drinks”) under fully dietary controlled conditions. Knee extensor total isokinetic work (“muscle function”) was measured in both legs at baseline (day 1), and 24 (day 8) and 48 (day 9) hours following a single bout of unilateral knee extensor ECs (day 7). Eccentric contractions were performed in 1 leg (“damaged”) randomly counterbalanced for leg dominance. The contralateral leg functioned as a control (“control”). To determine free-living myoPS rates of damaged and control legs, participants underwent a deuterated water dosing protocol, which began with a loading day on day 7 and continued with daily maintenance doses on days 8 and 9 (see “Deuterated Water”). Bilateral muscle biopsies of the *vastus lateralis* were taken 24, 27, and 36 hours following eccentric exercise and categorized into postexercise (24–27 hours) and overnight (27–36 hours) recovery periods. Myofibrillar protein synthesis rates and changes in skeletal muscle gene expression in damaged and control legs was determined for postexercise and overnight periods. Venous blood samples were collected on day 1 and 24, 27, 36, and 48 hours following eccentric exercise.

### Eccentric knee extensor contractions

At ~7:00 pm on day 7, participants performed 300 (10 sets of 30 repetitions of) voluntary maximal, unilateral, isokinetic, ECs of the knee extensors in the damaged leg—a volume of ECs previously shown to cause ultrastructural damage to myofibrillar structures (1). Each contraction was performed at 60^o^·sec^-1^ over an 80^o^ range of motion, which ended at full voluntary knee flexion. Each set was separated by 120 seconds of rest. Participants were instructed to resist the movement maximally throughout the full range of motion and were provided with verbal encouragement.

### Muscle soreness and muscle function

Muscle soreness and muscle function measurements began at 7:00 pm, 2 hours following ingestion of the controlled evening meal. Lower body muscle soreness was measured by asking participants to stand from a seated position and rate their corresponding sensation of lower body muscle soreness on a 100-mm visual analogue scale anchored by “no pain” at 0 mm and “worst possible pain” at 100 mm. Muscle function was calculated as the area under the torque-time curve following 30 maximal, concentric, isokinetic knee extensor contractions. Contractions were performed at 75^o^·sec^-1^ through an 80^o^ range of motion equidistant from voluntary maximal knee extension and flexion, and were preceded by a 5-repetition submaximal warm-up. Muscle function was determined first in the damaged leg followed by the control leg. On day 8 the damaged leg performed an additional 4 sets of 30 maximal concentric contractions separated by 60 seconds of rest (ie, 150 contractions in total), and the control leg performed as many maximal repetitions as necessary (in sets of 30) to match this volume of work. This was included to stimulate a postexercise protein synthetic response. Verbal encouragement was provided throughout all exercises.

### Skeletal muscle tissue collection

On day 8, participants arrived at the laboratory at 5:30 pm, 1.5 hours following ingestion of the controlled evening meal. First, muscle soreness was assessed. Next, bilateral muscle biopsy samples were obtained (ie, 24 hours following ECs) followed by muscle function testing and an additional work-matched bout of concentric exercise. Participants then ingested a postexercise drink according to their condition randomization and rested semisupine in the laboratory for a 3-hour postexercise period when a second set of bilateral muscle biopsies were sampled (ie, 27 hours following ECs). Participants then ingested a prebed drink according to their condition randomization and returned home to sleep, returning to the laboratory the following morning when a 3^rd^ set of bilateral muscle biopsies were sampled (ie, 36 hours following ECs) exactly 9 hours after ingestion of the prebed drink. No additional nutrition or physical activity was permitted during these periods. Muscle biopsies were collected from the midregion of the *vastus lateralis* (approximately 15 cm above the patella) with a modified Bergström needle under local anesthesia (2% lidocaine). All biopsy samples were immediately freed from any visible blood, adipose, and connective tissue, and were frozen immediately in liquid nitrogen-cooled isopentane and stored at -80°C until subsequent analysis. Successive biopsies were collected from a different incision, approximately 2 cm proximal to the previous incision, and the Bergström needle was angled to avoid sampling any tissue in close proximity to previous biopsies.

### Experimental drinks

Drinks were provided by the manufacturer (Beachbody LLC, Santa Monica, California, USA) in sachets containing a single serving, and sachets were labeled using a coding system to ensure double-blinding. Protein-polyphenol and PLA drinks were independently analyzed for macronutrient composition (Premier Analytical Services, High Wycombe, Buckinghamshire, UK) and polyphenol content (PP drinks only; Eurofins Food Testing, Wolverhampton, UK). The detailed nutritional content of a representative drink sample is displayed in Table 2. The postexercise PP drink contained a blend of whey protein isolate, pea protein isolate, and micellar casein protein sources and 650 mg of pomegranate extract. The prebed PP drink contained micellar casein protein and 480 mg of tart cherry extract. The postexercise and prebed PLA drinks were taste-, texture-, and energy-matched to their respective PP drinks by substituting protein with carbohydrate (maltodextrin). All drinks were prepared by mixing 1 serving (postexercise = 35.5 g, prebed = 28.5 g) with 225 mL of water, followed by an additional 50 mL of water to “wash” the bottle and ensure that all contents were consumed. Postexercise drinks were consumed in the laboratory under supervision at 8:00 pm on day 1 and days 8 and 9. On days 2 to 6, when participants were not required to attend the laboratory, participants were instructed to consume the drinks at home at 8:00 pm. Prebed drinks were consumed by the participant at home within 30 minutes of going to bed, except for day 8 when it was consumed in the laboratory under supervision. All drinks were well tolerated, consumed within the allotted time (ie, 5 minutes), and resulted in no reported adverse effects during or after the experimental period.

**Table 2.** Nutritional content of a representative postexercise and prebed placebo and protein-polyphenol drink

	PLA		PP	
	Postexercise	Prebed	Postexercise	Prebed
Macronutrients				
Protein (g/serving)	0.9	0.3	21.1	17.0
Fat (g/serving)	0.3	0.2	0.6	0.3
Carbohydrate (g/serving)	29.2	23.7	8.9	6.1
Fibre (g/serving)	2.3	2.6	1.2	2.4
Energy (kcal/serving)	127.8	103.2	127.8	99.5
Energy (kJ/serving)	541.7	437.2	542.1	421.5
Polyphenol content (GAE/serving)	n/a	n/a	211.2	78.4

Abbreviations: GAE, gallic acid equivalent; PLA, placebo; PP, protein-polyphenol.

### Dietary control

Habitual energy and macronutrient intakes were calculated from a 3-day food diary using online licensed software (Nutritics, Swords, Co, Dublin, Ireland). During the 9-day experimental period, participants received a fully controlled diet from the research team, which was weighed out and prepared in a metabolic kitchen. Participants received all food products in individual packets and received step-by-step recipes. All meals and snacks were provided, whereas water and noncaloric drinks were allowed ad libitum. Caffeinated drinks were permitted before midday only. Energy requirements were calculated as the basal metabolic rate (22) multiplied by an activity factor of 1.6. Daily protein intake was standardized at 1.2 g·kgBM^-1^·d^-1^ (~17% of energy intake), with the remaining calories being contributed by fat (~33% of energy intake) and carbohydrate (~50% of energy intake). Compliance with the nutritional intervention was assessed via the completed 9-day food diaries, returned food containers, and daily communication with the participants.

### Deuterated water

The deuterated water dosing protocol consisted of 1 loading day (day 7) and 2 maintenance days (days 8 and 9) to achieve and maintain 0.6% body water deuterium enrichment on days 8 and 9. On day 7 participants arrived at the laboratory overnight fasted at 6:00 pm and consumed a total of 6 mL·kgBM^-1^ of deuterated water (70 atom %; Cambridge Isotope Laboratories Inc, Tewksbury, MA, USA) in 8 doses spaced 1.5 hours apart. Participants were provided with breakfast after the first dose and remained in the laboratory until the 4^th^ dose was consumed (12:30 pm) and then returned home to consume the remaining 5 doses (ending at 6:30 pm ). Thereafter, participants consumed a daily maintenance dose of 0.54 mL·kgBM^-1^ 70% deuterated water upon waking on days 8 and 9.

### Blood sample collection and analysis

Ten mL of venous blood from the antecubital vein was collected on day 1 and 24, 27, 36, and 48 hours following ECs. Venous blood was collected into lithium heparin-containing tubes (BD vacutainer LH; BD Diagnostics, Nu-Care, Bedfordshire, UK) and centrifuged immediately at 3000 x g at 4°C for 10 minutes. Blood plasma was aliquoted and frozen at -80^o^C for subsequent analysis. Hydrogen isotope ratios (^2^H/^1^H) of plasma were determined in triplicate by injecting samples into a high-temperature conversion elemental analyzer (TCEA Flash 2000; ThermoFisher Scientific, Waltham, MA, USA) coupled to an isotope ratio mass spectrometer ([IRMS] Delta V; ThermoFisher Scientific). Raw isotope ratio values were normalized with in-house reference materials calibrated to Vienna Standard Mean Ocean Water.

### Myofibrillar-bound [^2^H]alanine enrichment

The enrichment of [^2^H]alanine in the myofibrillar fraction of skeletal muscle tissue samples was determined as described previously (16). Briefly, ~50 mg of whole frozen muscle was mechanically homogenized in 7.5 volumes of ice-cold homogenization buffer (Tris-HCL [pH 7.4] 50 mM, EDTA 1 mM, EGTA 1 mM, β-glycerophosphate 10 mM, NaF 50 mM, activated sodium orthovanadate 0.5 mM, and 1 complete mini protease inhibitor cocktail tablet per 50 mL of buffer [Roche Holding AG, Basel, Switzerland]). Homogenized samples were centrifuged (2200 x g at 4^o^C for 10 minutes) and the supernatant containing the sarcoplasmic protein fraction was removed and stored at -80^o^C for western blotting analysis (see “Western Blotting”). The remaining pellet was washed in 500 µL ice-cold homogenization buffer, centrifuged (700 x g at 4^o^C for 10 minutes), and solubilized (750 µL 0.3 M sodium hydroxide at 50^o^C for 30 minutes). After centrifugation (10 000 x g at 4^o^C for 10 minutes), myofibrillar proteins were precipitated from the supernatant by adding 500 µL of 1 M perchloric acid and vortexing for 30 seconds, and pelleted by centrifugation (700 x g at 4^o^C for 10 minutes). The pellet was washed twice in 70% ethanol and amino acids were hydrolyzed in 2 mL of 6 M hydrochloric acid at 110^o^C for 24 hours. The samples were subsequently dried under a vacuum (Savant SpeedVac; ThermoFisher Scientific), reconstituted in 3 mL 25% acetic acid, passed over cation exchange resin columns (100–200 mesh, H^+^ form, Dowex 50WX8; Sigma-Aldrich Company Ltd, Gillingham, UK) and eluted with 6 M NH_4_OH before being dried again under vacuum. Samples were resuspended in 1 mL distilled water and 1 mL 0.1% formic acid in acetonitrile, centrifuged (10 000 x g at 4°C for 3 minutes), and the supernatant was aliquoted, dried under a vacuum, and stored at -20°C.

Amino acids were derivatized by adding 50 μL N-tert-Butyldimethylsilyl-N-methyltrifluoroacetamide + 1% tert-butyl-dimethylchlorosilane and 50 μL acetonitrile, and vortexed and heated at 95°C for 40 minutes. The samples were then transferred to a gas chromatography vial. Alanine enrichment was analyzed using a ThermoFisher Delta V Advantage IRMS (Bremen, Germany) fitted with a Trace 1310 gas chromatograph with an online high-temperature thermal conversion oven at 1420°C. The sample (1 μL) was injected in splitless mode at an injection port temperature of 250°C. The peaks were resolved on a 30 m × 0.25 mm ID × 0.25 μm film Agilent Technologies DB-5 capillary column (temperature program: 110°C for 1 minute; 10°C∙min^-1^ ramp to 180°C; 5°C∙min^-1^ ramp to 220°C; 20°C∙min^-1^ ramp to 300°C; hold for 2 minutes) prior to pyrolysis. Helium was used as the carrier gas, with a constant flow of 1 mL/minute. Any amino acid eluting from the gas chromatograph was converted to H_2_ prior to entry into the IRMS.

The enrichment of tracer was measured by monitoring ion masses 2 and 3 to determine the 2H/1H ratios of myofibrillar protein-bound [2H]alanine. A series of known standards were applied to assess both the linearity of the mass spectrometer and to control for the loss of tracer.

### Western blotting

Skeletal muscle total and phosphorylated (mTOR^Ser2448^) forms of mTOR were determined as described previously (16). Briefly, the protein content of the sarcoplasmic fraction of skeletal muscle samples was determined by colorimetric assay (DC protein assay; Bio-Rad Laboratories, Inc, CA, USA). Proteins were denatured by incubating for 5 minutes at 95°C in XT sample buffer (Bio-Rad Laboratories). Twenty µg protein per lane was loaded onto 3%–8% tris-acetate polyacrylamide gels, and separated by electrophoresis in XT tricine running buffer for 65 minutes at 150 V. Proteins were transferred to 0.2 µM nitrocellulose membranes using a trans-blot turbo transfer system (Bio-Rad Laboratories) at 2.5 A and 25 V for 10 minutes. Membranes were blocked in 5% BSA in Tris-Buffered Saline (TBST) (pH 7.6) for 1 hour before overnight incubation at 4°C with rabbit antiphospho-mTOR monoclonal antibody (5536, 1:1000 in TBST; Cell Signaling Technology, Danvers, MA, USA) and rabbit anti-α-tubulin (11H10, 1:20000 in TBST; Cell Signaling Technology, Inc) loading control. Following 3 10-minute washes in TBST, membranes were incubated for 1 hour at room temperature in secondary horseradish perioxidase (HRP) conjugated anti-rabbit IgG antibody (ab6721, 1:3000 in TBST; Abcam PLC, Cambridge, UK). Following 3 10-minute washes in TBST, membranes were then exposed for 5 minutes in Clarity Western chemiluminescent detector solution (Bio-Rad Laboratories), visualized using a Chemidoc scanner (Bio-Rad Laboratories), and band density quantified using Image Lab software (Bio-Rad Laboratories). The expected migration of mTOR^Ser2448^ (~289 kDa) and α-tubulin (~52 kDa) was confirmed using a kaleidoscope protein ladder (Bio-Rad Laboratories). For total mTOR, membranes were incubated for 15 minutes in Restore stripping buffer (Thermo Fisher Scientific, Waltham, MA, USA), blocked for 1 hour in 5 % BSA in TBST and re-probed overnight with an anti-mTOR monoclonal primary antibody (2972, 1:1000 in TBST; Cell Signaling Technology) plus anti-α-tubulin, and the above steps were repeated to obtain corresponding bands for total mTOR. The band density for phospho-mTOR was calculated as a ratio against the band density for α-tubulin, within each lane. This was divided by the ratio of mTOR against α-tubulin to give an overall ratio for mTOR phosphorylation status.

### Gene expression

Total ribonucleic acid (RNA) was extracted from ~20 mg frozen muscle tissue using TRI Reagent (Thermo Fisher Scientific) (23), according to the manufacturer’s protocol. Total RNA quantification was carried out spectrophotometrically at 260 nm (NanoDrop ND-2000 Spectrophotometer; Thermo Fisher Scientific) and RNA purity was determined as the ratio of readings at 260/280 nm. Reverse transcription of RNA was carried out using a commercially available kit (SuperScript VILO cDNA Synthesis Kit; Thermo Fischer Scientific). Thereafter, relative gene expression of 224 genes (Supplementary Table S1(24)) from pathways associated with cellular amino acid transport, extracellular matrix remodeling, inflammation, insulin signaling, myogenesis, protein turnover, stress response, substrate metabolism, and associated nuclear transcription factors were determined using custom-designed quantitative reverse transcription PCR (RT-qPCR) OpenArray plates (Thermo Fischer Scientific) in combination with a QuantStudio 12K Flex Real-Time PCR system (Thermo Fischer Scientific). The candidate genes were selected from PubMed literature searches, pathway databases, and data from our laboratories. The threshold cycle C_T_ value was automatically calculated by the QuantStudio 12k Flex software. Relative gene expression was calculated using the 2^-ΔΔCT^ method, with the geometric mean of the reference genes α-actin, β-actin, and β2 microglobulin used for normalization purposes. A pooled internal calibrator sample was used to account for plate-to-plate variability. Expression levels in the damaged leg were calculated separately at each timepoint using the control leg as the comparator and subsequently log_2_ transformed to ensure normal distribution of the data. A total of 213 genes were included in the final analysis. Exploratory functional enrichment analysis of significant genes was performed against the Reactome database using the PANTHER overrepresentation test (binomial test with no correction; released 20190711) (PANTHER version 15.0). The original gene list, as opposed to the whole genome, was used as the background. Twenty-one genes loaded onto the OpenArray plates associated with NF-κB activation (9 upstream and 12 downstream) and previously shown to be upregulated in response to ECs were selected *a priori* for a targeted analysis (8).

### Calculations

Myofibrillar protein fractional synthesis rates were calculated based on the incorporation of [^2^H]alanine into myofibrillar protein and mean plasma deuterium enrichment between muscle biopsy timepoints using the following standard precursor-product equation:

$$\begin{array}{l} MyoPS\;\left(\%\cdot h^{-1}\right)=\left[\frac{\Delta Ep}{Eprecursor\times t}\right]\times 100 \end{array}$$

where Δ*E*p is the increment in [^2^H]alanine enrichment in myofibrillar protein between 2 biopsies, *E*_precursor_ is the average body water deuterium enrichment between 2 biopsies corrected by a factor of 3.7 based upon the deuterium labeling of alanine during *de novo* synthesis, and *t* indicates the tracer incorporation time between 2 muscle biopsies.

### Statistics

Differences in subject characteristics and dietary intake between conditions were analyzed using unpaired t-tests. A repeated-measure 2-factor analysis of variance (ANOVA) was used to analyze (condition *x* time) differences in body water ^2^H enrichment, muscle soreness, muscle function calculated as a percentage of the control leg and NF-κB gene expression, and (condition *x* leg) differences in myoPS rates. Repeated-measure 3-factor ANOVA was used to analyze (time *x* condition *x* leg) differences in myofibrillar mole percent excess (MPE), postexercise vs overnight myoPS rates, total mTOR, mTOR^Ser2448^, and mTOR phosphorylation status. A repeated-measure mixed-effects model was used to analyze (condition *x* time) differences in postexercise and overnight gene expression. A false discovery rate (FDR < 0.05) was applied to adjust for family-wise error prior to functional enrichment analysis (25). Participant sex was not considered a factor in the statistical analysis of the data. Violations of sphericity were corrected with the Greenhouse-Geisser correction. When a significant main and interaction effect was observed, a Sidak post hoc test was performed to locate individual differences. Statistical significance was set at *P* < 0.05. Calculations were performed using GraphPad Prism 8.3.0. All data are expressed as means ± standard error of the mean (SEM).

## Results

### Participant characteristics and diet

No differences in age, weight, height, BMI, or habitual protein intake were detected between conditions (*P* > 0.05) (Table 1). Average daily energy and macronutrient ingestion during the 9-day controlled diet after accounting for adherence (not including condition) was 10775 ± 279 KJ with 52 ± 1% energy as carbohydrate, 33 ± 1% energy as fat, and 13 ± 1% energy (1.2 ± 0.1 g·kgBM^-1^·d^-1^) as protein, and was not different between conditions (*P* > 0.05). Protein-polyphenol ingestion increased protein intake compared with PLA (1.8 ± 0.1 vs 1.2 ± 0.1 g·kgBM^-1^·d^-1^, respectively; *P* < 0.001). Total eccentric work performed did not differ between conditions (*P* > 0.05). Body water deuterium enrichment and skeletal muscle analysis were performed in *n* = 17 unless otherwise stated, as 1 participant (female, PLA) completed the study without muscle biopsies.

### Muscle soreness and function

**Figure 2.**
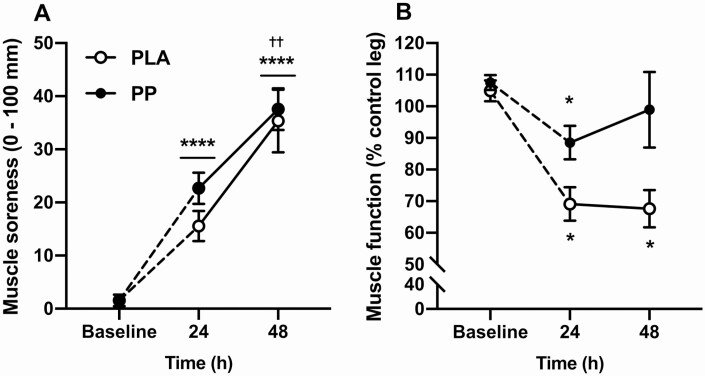
**A**: Muscle soreness upon standing from a seated position measured using 100 mm visual analogue scale anchored by “no pain at all” (0 mm) to “worst possible pain” (100 mm). **B**: Knee extensor total isokinetic work (“muscle function”) performed during 30 maximal, isokinetic, concentric knee extensor contractions expressed relative to the contralateral control leg. Measurements were taken at baseline and 24 and 48 hours following 300 unilateral eccentric knee extensor contractions (time = 0 hours). Postexercise and prebed protein-polyphenol (*n* = 9; black circles) or isocaloric maltodextrin placebo (*n* = 9; open circles) drinks were ingested for 6 days prior to, and 3 days following, eccentric contractions. Data are presented as means with error bars representing standard error. Statistical analysis was performed with separate 2-factor analysis of variance. *Timepoint different to baseline; ^†^timepoint different to 24 hours. One symbol, *P* < 0.05; 2 symbols, *P* < 0.01; 3 symbols, *P* < 0.001; 4 symbols, *P* < 0.0001. Horizontal bars indicate main effects, whereas individual symbols represent post hoc effects.

Note: Figure Replacement Requested.

Three hundred maximal voluntary ECs increased muscle soreness from baseline (*P* < 0.001) after 24 hours (baseline = 1.6 ± 0.7, 24 hours = 19.1 ± 2.2 mm; post hoc; *P* < 0.001), which increased further after 48 hours (48 hours = 36.4 ± 3.5 mm; post hoc; *P* = 0.003; Fig. 2A). Muscle soreness was not different between conditions (*P* = 0.215, interaction; *P* = 0.603). Eccentric contractions impaired muscle function relative to baseline (*P* < 0.001), but the impairment was attenuated with PP compared with PLA ingestion (interaction; *P* = 0.047; Fig. 2B). With PLA ingestion, muscle function decreased from baseline by 36 ± 7% after 24 hours (post hoc; *P* = 0.004) and plateaued at 37 ± 7% below baseline after 48 hours (post hoc; *P* = 0.002). With PP ingestion, muscle function was decreased from baseline only after 24 hours (19 ± 6% post hoc; *P* = 0.036) and had recovered to baseline after 48 hours (9 ± 12%; post hoc; *P* = 0.879). The impairment in muscle function after 24 hours also tended to be attenuated with PP, relative to PLA, ingestion (post hoc; *P* = 0.057).

### Body water deuterium enrichment

**Figure 3.**
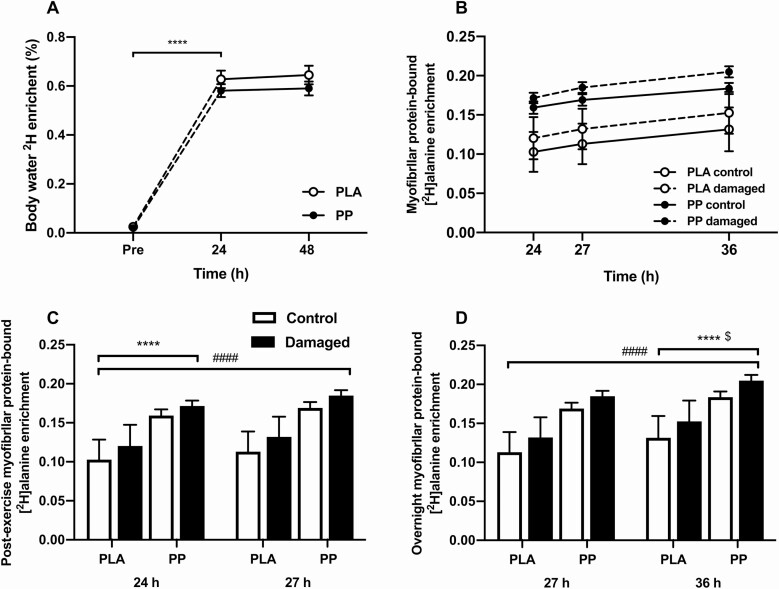
**A**: Body water ^2^H enrichment (%) measured in blood plasma at baseline and 24 and 48 hours following 300 unilateral eccentric knee extensor contractions (time = 0 hours). **B**: Myofibrillar bound [^2^H]alanine enrichment relative to a linear scale on the x-axis to demonstrate the linear increase in enrichment with respect to time in a control (solid line) and eccentrically exercised (dashed line) leg. Postexercise and prebed protein-polyphenol (PP; *n* = 9; black circles) or isocaloric maltodextrin placebo (PLA; *n* = 9; open circles) drinks were ingested for 6 days prior to, and 3 days following, eccentric contractions. **C–D**: Postexercise (24 vs 27 hours) and overnight (27 vs 36 hours) enrichment of myofibrillar-bound [^2^H]alanine (MPE) in skeletal muscle biopsy samples in a control (white bars) and eccentrically exercised (black bars) leg. Postexercise and prebed PP (*n* = 9) or isocaloric maltodextrin PLA (*n* = 9) drinks were ingested for 6 days prior to, and 3 days following, eccentric contractions (time = 0 hours). Data are presented as means, with error bars representing standard error. Body water ^2^H nrichment was analyzed with a 2-factor analysis of variance (ANOVA). Postexercise and overnight myofibrillar-bound [^2^H]alanine enrichment were analyzed with separate 3-factor ANOVA. *Timepoint different to 27 hours; ^#^damaged leg different to control leg; ^$^the increase in MPE (27 vs 36 hours) is different between the damaged and control legs. One symbol, *P* < 0.05; 2 symbols, *P* < 0.01; 3 symbols, *P* < 0.001; 4 symbols, *P* < 0.0001.

Baseline body water deuterium enrichment did not differ between PLA (0.03 ± 0.01%) and PP (0.02 ± 0.01%) (*P* > 0.05; Fig. 3A). Deuterated water loading increased body water deuterium enrichment (*P* < 0.001) to a similar extent in PLA and PP (to 0.63 ± 0.04% and 0.58 ± 0.03%, respectively; interaction; *P* = 0.363). Body water deuterium enrichment did not change during the 2 days of maintenance dosing (*P* = 0.233), averaging 0.64 ± 0.03% in PLA and 0.59 ± 0.02% in PP, with no difference between conditions (*P* = 0.267).

### Myofibrillar protein-bound [^2^H]alanine enrichment and myoPS rates

Myofibrillar protein-bound [^2^H]alanine enrichments increased during the postexercise (Fig. 3C) and overnight (Fig. 3D) incorporation periods (*P* < 0.001). The increase in postexercise myofibrillar [^2^H]alanine enrichment tended to be greater in the damaged leg (control = ∆0.010 ± 0.001 MPE, damaged = ∆0.012 ± 0.001; interaction; *P* = 0.07), and the increase in overnight myofibrillar [^2^H]alanine enrichment was greater in the damaged leg (control = ∆0.016 ± 0.002 MPE, damaged = ∆0.020 ± 0.002; interaction; *P* = 0.042). There were no condition effects on the increase in MPE during postexercise (interaction; *P* = 0.706) and overnight (interaction; *P* = 0.493) incorporation periods.

**Figure 4.**
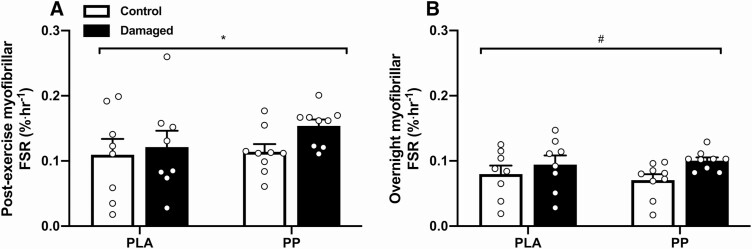
Myofibrillar protein fractional synthesis rate (FSR; expressed as %·h^-1^) over a 3-hour postexercise period following a work-matched bout of maximal concentric knee extensor contractions and ingestion of either a postexercise protein-polyphenol (PP; *n* = 9) or isocaloric maltodextrin placebo (PLA; *n* = 9) drink (**A**), and the subsequent 9-hour overnight period, beginning with ingestion of either a prebed PP (*n* = 9) or isocaloric maltodextrin PLA (*n* = 9) drink (**B**). Postexercise and overnight periods correspond to between 24 and 27 hours, and 27 and 36 hours, respectively, following 300 maximal unilateral eccentric knee extensor contractions (damaged, black bars), while the contralateral leg rested (control, white bars). FSRs are calculated from body water deuterium enrichment as precursor pool. Data are presented as means, with individual responses shown with open circles (○). Statistical analysis was performed with separate 2-factor analysis of variance (ANOVA) and the postexercise vs overnight period was compared with a 3-factor ANOVA. *Difference between postexercise and overnight periods; ^#^difference between damaged and control legs. One symbol, *P* < 0.05.

Postexercise myoPS rates following a work-matched bout of concentric exercise tended to be higher in the damaged leg than in the control leg (0.14 ± 0.01 vs 0.11 ± 0.01%·h^-1^, respectively; *P* = 0.075; Fig. 4A). Postexercise myoPS rates were not affected by condition (*P* = 0.412, interaction; *P* = 0.313). Myofibrillar protein synthesis rates decreased (*P* = 0.027) from the postexercise to the overnight period similarly between control (from 0.11 ± 0.01 to 0.07 ± 0.01%·h^-1^, respectively) and damaged (from 0.14 ± 0.01 to 0.10 ± 0.01%·h^-1^, respectively) legs (interaction; *P* = 0.820). Overnight myoPS rates were higher in the damaged legs compared with the control legs (0.10 ± 0.01 vs 0.07 ± 0.01%·h^-1^, respectively; *P* = 0.020) but were not affected by condition (*P* = 0.908, interaction; *P* = 0.385; Fig. 4B).

### mTOR protein content and phosphorylation status

Western blotting was performed in *n* = 16 (PLA = 8, PP = 8) due to limited remaining tissue.

#### Postexercise

**Figure 5.**
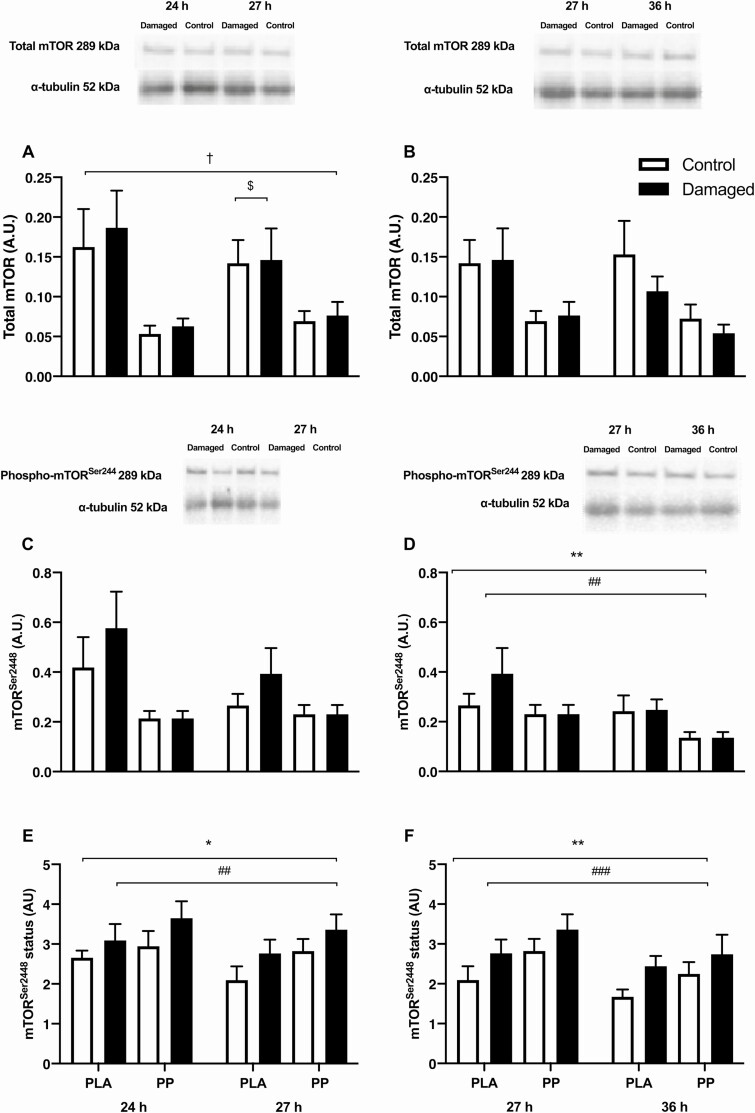
Skeletal muscle mechanistic target of rapamycin (mTOR) phosphorylation status, presented as total mTOR protein during postexercise (**A**) and overnight (**B**) periods; mTOR phosphorylated at Ser2448 during postexercise (**C**) and overnight (**D**) periods; and the ratio of phosphorylated to total protein during postexercise (**E**) and overnight (**F**) periods following 300 unilateral eccentric knee extensor contractions (time = 0 hours) (damaged; black bars), while the contralateral leg rested (control; white bars). The postexercise period incorporated a work-matched bout of maximal concentric knee extensor contractions and ingestion of either a postexercise protein-polyphenol (PP; *n* = 9) or isocaloric maltodextrin placebo (PLA; *n* = 9) drink. The subsequent 9-hour overnight period began with ingestion of either a prebed PP (*n* = 9; black bars) or isocaloric maltodextrin PLA (*n* = 9; white bars) drink. α-tubulin was used as a loading control. Images obtained from a single representative participant in PLA. Data are presented as means, with error bars representing standard error. Statistical analysis was performed with separate 2-factor analysis of variance. *Main effect of time; ^#^difference between damaged and control legs; ^†^difference between PLA and PP; ^$^decrease between 24 and 27 hours in PLA only. One symbol, *P* < 0.05; 2 symbols, *P* < 0.01; 3 symbols, *P* < 0.001.

Skeletal muscle total mTOR protein content did not change during the postexercise period (*P* = 0.448; Fig. 5A) and was not different between control and damaged legs (*P* = 0.119). Postexercise total mTOR protein content was higher in PLA than PP (*P* = 0.038) and decreased similarly between legs in PLA (post hoc; *P* = 0.031) during the postexercise period but did not change in PP (post hoc; *P* = 0.392). Skeletal muscle mTOR^Ser2448^ protein content did not change during the postexercise period (P = 0.127; Fig. 5C) and tended to be higher in the damaged leg compared with the control leg (*P* = 0.053), tended to be higher in PLA compared with PP (*P* = 0.070), and tended to interact between legs and conditions (interaction; *P* = 0.068). Skeletal muscle mTOR^2448^ phosphorylation status decreased by 7 ± 5% during the postexercise period (*P* = 0.035; Fig. 5E) and was 19 ± 8% higher in the damaged leg compared with the control leg (*P* = 0.002). Postexercise mTOR^2448^ phosphorylation status was not different between conditions (*P* = 0.230, interaction effect; *P* = 0.496).

### Overnight

Skeletal muscle total mTOR protein content did not change during the overnight period (*P* = 0.133; Fig. 5B) and tended to be different between legs (*P* = 0.059) and conditions (*P* = 0.057), but condition and leg did not interact during the overnight period (interaction; *P* = 0.617). Overnight skeletal muscle mTOR^Ser2448^ protein content decreased during the overnight period (*P* = 0.006; Fig. 5D) and was higher in the damaged leg than in the control leg (*P* = 0.009). Overnight mTOR^Ser2448^ protein content was not different between conditions (*P* = 0.104, interaction; *P* = 0.390). Skeletal muscle mTOR^2448^ phosphorylation status decreased by 15 ± 4% during the overnight period (*P* = 0.001; Fig. 4F) and was 25 ± 7% higher in the damaged leg compared with the control leg (*P* < 0.001). Postexercise mTOR^2448^ phosphorylation status was not different between conditions (*P* = 0.222, interaction; all *P* = 0.775).

### Targeted NF-κB–associated transcription

Gene expression was calculated for *n* = 15 (PLA = 7, PP = 8) due to limited remaining tissue.

#### Postexercise

**Figure 6.**
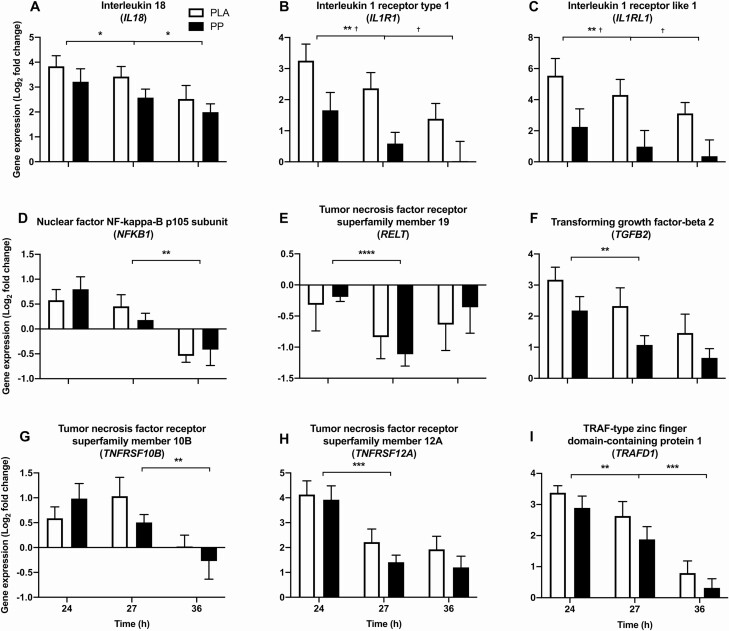
Skeletal muscle mRNA expression of 9 genes (**A**–**I**) upstream of NF-κB activity selected a priori for targeted analysis. Gene expression was measured during postexercise (24–27 hours) and overnight (27–36 hours) periods, following 300 unilateral eccentric knee extensor contractions (time = 0 hours). Postexercise and prebed protein-polyphenol (PP; *n* = 9; black bars) or isocaloric maltodextrin placebo (PLA; *n* = 9; white bars) drinks were ingested for 6 days prior to, and 3 days following, eccentric contractions. All individual expression values are expressed as a fold-change from the contralateral control leg at the same timepoint, with log_2_ transformation applied. Data are presented as means, with error bars representing standard error. Statistical analysis was performed with separate 2-factor analysis of variance. *Main effect of time; ^†^difference between PLA and PP. One symbol, *P* < 0.05; 2 symbols, *P* < 0.01; 3 symbols, *P* < 0.001; four symbols, *P* < 0.0001.

**Figure 7.**
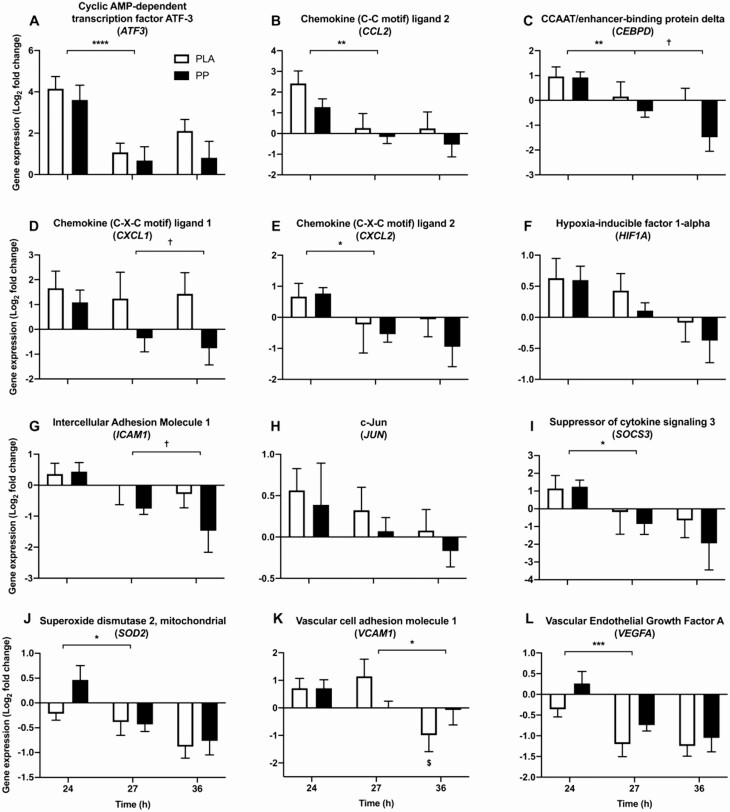
Skeletal muscle mRNA expression of 12 genes (**A**–**L**) downstream of NF-κB activity selected a priori for targeted analysis. Gene expression was measured during postexercise (24–27 hours) and overnight (27–36 hours) periods, following 300 unilateral eccentric knee extensor contractions (time = 0 hours). Postexercise and prebed protein-polyphenol (PP; *n* = 9; black bars) or isocaloric maltodextrin placebo (PLA; *n* = 9; white bars) drinks were ingested for 6 days prior to, and 3 days following, eccentric contractions. All individual expression values are expressed as a fold-change from the contralateral control leg at the same timepoint, with log_2_ transformation applied. Data are presented as means, with error bars representing standard error. Statistical analysis was performed with separate 2-factor analysis of variance. *Main effect of time; ^†^difference between PLA and PP; ^$^decease in expression in PLA only. One symbol, *P* < 0.05; 2 symbols, *P* < 0.01; 3 symbols, *P* < 0.001; 4 symbols, *P* < 0.0001.

Note: Figure Replacement Requested.

The expression of 7 upstream (interleukin 18 [*IL18*], interleukin 1 receptor type 1 [*IL1R1*], interleukin 1 receptor-like 1 [*IL1RL1*], tumor necrosis factor receptor superfamily member 19 [*RELT*], transforming growth factor-beta 2 [*TGFB2*], tumor necrosis factor receptor superfamily member 12A [*TNFRSF12A*], and TRAF-type zinc finger domain-containing protein 1 [*TRAFD1*]; *P* < 0.05, Fig. 6) and 7 downstream (cyclic AMP-dependent transcription factor ATF-3 [*ATF3*], chemokine [C-C motif] ligand 2 [*CCL2*], CCAAT/enhancer-binding protein delta [*CEBPD*], chemokine (C-X-C motif) ligand 2 [*CXCL2*], suppressor of cytokine signaling 3 [*SOCS3*], superoxide dismutase 2, mitochondrial [SOD2], and vascular endothelial growth factor A [*VEGFA*]; *P* < 0.05, Fig. 7) genes of NF-κB activation were differentially expressed during the postexercise period. NF-κB p105 subunit (*NFKB1*) (upstream, *P* = 0.065) and hypoxia-inducible factor 1 subunit alpha (*HIF1A*) (downstream, *P* = 0.079) tended to be downregulated during the postexercise period. Relative to PLA, PP ingestion downregulated the postexercise expression of *IL1R1* (upstream, mean postexercise log_2_ fold-change; PLA = 2.81 ± 0.37, PP = 1.12 ± 0.36; *P* = 0.024, Fig. 6B) and *IL1RL1* (upstream, mean postexercise log_2_ fold-change; PLA = 4.92 ± 0.74, PP = 1.61 ± 0.77; *P* = 0.044, Fig. 6C). Protein-polyphenol ingestion also tended to downregulate *TGFB2* expression (*P* = 0.072) and attenuate the downregulation of *VEGFA* (*P* = 0.064). No NF-κB–associated genes interacted between conditions over the course of the postexercise period, but tumor necrosis factor receptor superfamily member 10B (*TNFRSF10B*) showed a tendency to (*P* = 0.078).

### Overnight

Five NF-κB–associated genes were downregulated during the overnight period of which 4 genes were upstream (*IL18, TRAFD1, NFKB1 and TNFRSF10B; P* < 0.05, Fig. 6) and 1 gene was downstream (vascular cell adhesion molecule 1 [*VCAM1*]*; P* < 0.05, Fig. 7) of NF-κB activation. Relative to PLA, PP ingestion further downregulated the overnight expression of *IL1R1* (upstream, mean overnight log_2_ fold-change; PLA = 1.87 ± 0.37, PP = 0.31 ± 0.36; *P* = 0.018, Fig. 6B) and *IL1RL1* (upstream, mean overnight log_2_ fold-change; PLA = 3.71 ± 0.61, PP = 0.67 ± 0.72; *P* = 0.025, Fig. 6C). Protein-polyphenol ingestion also downregulated the overnight expression of chemokine (C-X-C motif) ligand 1 (*CXCL1*) (downstream, mean overnight log_2_ fold-change; PLA = 1.34 ± 0.65, PP = -0.56 ± 0.42; *P* = 0.028, Fig. 7D), *CEBPD* (downstream, mean overnight log_2_ fold-change; PLA = 0.09 ± 0.36, PP = -0.96 ± 0.32; *P* = 0.010, Fig. 7C), and intercellular adhesion molecule 1 (*ICAM1*) (downstream, mean overnight log_2_ fold-change; PLA = -0.15 ± 0.37, PP = -1.11 ± 0.36; *P* = 0.044, Fig. 7G) relative to PLA. *VCAM1* (downstream) was downregulated with PLA ingestion (from 1.14 ± 0.62 at 27 hours to -0.99 ± 0.60 at 36 hours) but did not change with PP ingestion (from 0.17 ± 0.20 at 27 hours to -0.33 ± 0.55 at 36 hours) (interaction; *P* = 0.041, Fig. 7K).

### Exploratory functional gene enrichment analysis

**Figure 8.**
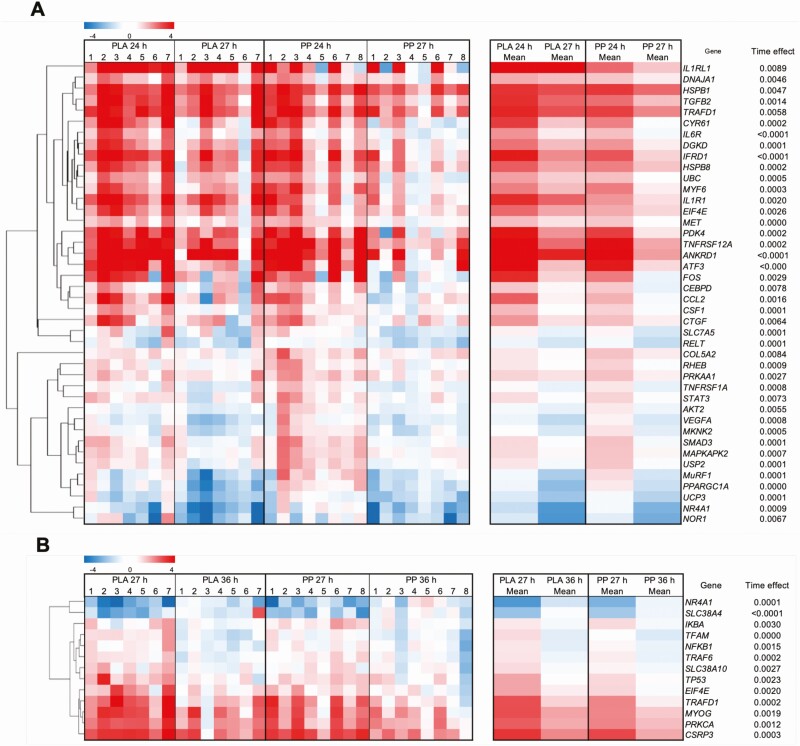
Skeletal muscle mRNA expression of genes that were differentially expressed during the postexercise (24–27 hours; **A**) or overnight (27–36 hours; **B**) period (time effect; FDR < 5%) following 300 unilateral eccentric knee extensor contractions (time = 0 hours). Postexercise and prebed protein-polyphenol (PP; *n* = 8) or isocaloric maltodextrin placebo (PLA; *n* = 7) drinks were ingested for 7 days prior to, and 1 day following, eccentric contractions. All individual expression values are expressed as a fold-change from the contralateral control leg at the same timepoint, with log_2_ transformation and hierarchal clustering applied. Statistical analysis was performed with separate 2-factor mixed-effect models and corrected for familywise errors using a Benjamini-Hochberg correction. Abbreviation: FDR, false discovery rate.

The mRNA expression of all 213 genes included in the functional enrichment analysis is shown in Supplementary Table S1(24). No genes that were differentially expressed between conditions (ie, *P* < 0.05) passed the FDR cutoff of 5%. Thus, only genes that were expressed differentially over the postexercise and overnight period (ie, time effect) were analyzed for functional enrichment and are displayed in heat maps in Fig. 8A and 8B.

#### Postexercise

The expression of 42 genes was downregulated during the postexercise period, and no genes were upregulated (all significant using an FDR cutoff of *5*%, Fig. 8, Supplementary Table S1(24). This gene list did not significantly associate with any pathways in Reactome. Thirteen genes (V-AKT murine thymoma viral oncogene homolog 2 [*AKT2*], *CEBPD*, collagen type V alpha 2 chain [*COL5A2*], MAPK interacting serine/threonine kinase 2 [*MKNK2*], tripartite motif-containing 63 [*MURF1*], nuclear receptor subfamily 4 group A member 3 [*NOR1*], peroxisome proliferator-activated receptor gamma coactivator 1-alpha [*PPARGC1A*], AMP-activated, alpha 1 catalytic subunit [*PRKAA1*], *RELT*, solute carrier family 7 member 5 [*SLC7A5*], tumor necrosis factor receptor 1 [*TNFRSF1A*], uncoupling protein 3 [*UCP3*], and *VEGFA*) were downregulated exclusively postexercise. This gene list associated with FOXO-mediated transcription (*AKT2*, *MURF1*, and *PPARGC1A*; *P* = 0.046) in Reactome. Fourteen genes were differentially expressed between conditions (acetyl-CoA carboxylase beta [*ACACB*], *AKT2*, complement component 5a [*C5*], calpain 3 [*CAPN3*], CCAAT/enhancer-binding protein beta [*CEBPB*], CASP8 and FADD-like AP regulator [*CFLAR*], eukaryotic translation initiation factor 4E binding protein 1 [*EIF4EBP1*], hexokinase 2 [*HK2*], heat shock factor 4 [*HSF4*], interleukin 10 receptor, beta subunit [*IL10RB*], *IL1R1*, *IL1RL1*, paired box 7 [*PAX7*], and *PPARGC1A*; *P* < 0.05) and 7 genes were differentially expressed between conditions over the postexercise period (ras homolog family member B [*RHOB*], solute carrier family 38 member 2 [*SLC38A2*], transcription factor A, mitochondrial [*TFAM*], TNF receptor-associated factor 6 [*TRAF6*], tripartite motif containing 32 [*TRIM32*], tyrosine kinase 2 [*TYK2*], and ubiquitin B [*UBB*]; interaction; *P* < 0.05). However, these genes did not meet the FDR cutoff of 5% (Supplementary Table S1(24)).

### Overnight

The expression of 11 genes was downregulated, and the expression of 2 genes was upregulated during the overnight period (all significant using an FDR cutoff of *5*%, Fig. 8, Supplementary Table S124). Downregulated genes associated with 63 pathways in Reactome, of which TRAF6 mediated NF-κB activation (NF-κ light polypeptide gene enhancer in B-cells inhibitor, alpha [*IKBA*], *NFKB1*, and TNF receptor-associated factor 6 [*TRAF6*]; *P* = 0.003), NF-κB is activated and signals survival (*IKBA*, *NFKB1*, and *TRAF6*; *P* = 0.004), and TAK1 activates NF-κB by phosphorylation and activation of IKKs complex (*IKBA*, *NFKB1*, *TRAF6*; *P* = 0.009) were the 3 most enriched. The 2 upregulated genes were solute carrier family 38 member 4 (*SLC38A4;* amino acid transport, upregulated overnight exclusively) and nuclear receptor subfamily 4, group A, member 1 (*NR4A1;* transcription factor, downregulated postexercise and upregulated overnight). Nine genes were downregulated exclusively overnight (cysteine and glycine rich protein 3 [*CSRP3*], *IKBA*, myogenin [*MYOG*], *NFKB1*, protein kinase C alpha [*PRKCA*], solute carrier family 38 member 10 [*SLC38A10*], *TFAM*, tumor protein P53 [*TP53*], and *TRAF6*). This gene list associated 69 pathways in Reactome, of which TRAF6-mediated NF-κB activation (*IKBA*, *NFKB1*, and *TRAF6*; *P* = 0.002), NF-κB is activated and signals survival (*IKBA*, *NFKB1*, and *TRAF6*; *P* = 0.003), and TAK1 activates NF-κB by phosphorylation and activation of IKKs complex (*IKBA*, *NFKB1*, and *TRAF6*; *P* = 0.007) were the 3 most enriched. Nine genes were differentially expressed between conditions (calpastatin [*CAST*], *CEBPD*, *CXCL1*, C-Fos [*FOS*], *HK2*, heat shock protein family A [Hsp70] member 5 [*HSPA5*], *IL1R1*, *IL1RL1*, and *PPARGC1A*; *P* < 0.05) and 3 genes interacted between conditions over the course of the overnight period (caspase 9 [*CASP9*], myostatin [*MSTN*], *VCAM1*; interaction; *P* < 0.05). However, these genes did not meet the FDR cutoff of 5% (Supplementary Table S1(24)).

## Discussion

We have previously shown that postexercise PP ingestion increased overnight myoPS rates and accelerated the recovery of muscle function following damaging ECs (16). Presently, we further targeted overnight myoPS rates with a unique postexercise and prebed PP nutrition approach to determine if overnight myoPS rates underpin functional recovery. Remarkably, PP ingestion accelerated recovery of muscle function by 24 hours relative to PLA ingestion and, consistent with our previous work, ECs increased overnight myoPS rates by 30% compared with the control leg. However, the marked acceleration in functional recovery with PP ingestion observed presently was not paralleled by potentiated overnight myoPS rates. A major novel finding of the present study is that PP ingestion resulted in a coordinated downregulation in upstream and downstream skeletal muscle gene expression indicative of downregulated inflammatory IL-1β-mediated NF-κB activation. This immediately preceded the recovery of muscle function, suggesting inflammatory NF-κB transcriptional signaling could delay the recovery of muscle function following damaging ECs.

Isolated protein ingestion has been shown to accelerate the recovery of muscle function after ECs but, typically, baseline muscle function remains impaired for at least 48 hours with protein ingestion (14, 26–29). For example, Draganidis et al (14) reported that knee extensor function did not return to baseline until 72 hours following 300 ECs when 20 g of milk protein (80 g on the day of ECs) was ingested daily. Moreover, the efficacy of cherry or pomegranate phenolic extracts on accelerating recovery of knee extensor function when ingested in isolation is equivocal, with the currently limited evidence demonstrating positive (20, 30) or no effects (31). Presently, ECs caused an initial (24 hours) and sustained an (48 hours) ~37% decrease in muscle function with PLA ingestion. Remarkably however, postexercise and prebed PP ingestion attenuated this large initial decrease in muscle function by ~50% and restored baseline muscle function within 48 hours (Fig. 2B). This is consistent with the response we have previously shown in a separate study when PPs were ingested post-exercise only (16). Accordingly, targeting nutrient composition and providing a synergistic combination of protein and polyphenols within a few hours after eccentric exercise appears to be more efficacious at accelerating the rate of functional recovery than when protein or polyphenols are ingested in isolation. We did not perform a direct measure of myofibrillar injury in the current investigation. Whilst the ~37% decline in muscle function is well aligned with the decline in muscle function associated with myofibrillar damage after ECs (1), myofibrillar damage is not universally observed after ECs (32), so caution should be taken when translating these findings to injury.

Deuterated water can be used to sensitively determine myoPS rates over a period of hours (33), and in the present study it allowed overnight myoPS rates to be assessed while participants slept unrestricted in their own homes. Using deuterated water, we clearly demonstrate ECs increase free-living myoPS rates in the 24 to 36 hours postcontraction period (Fig. 4). Compared with the control leg, myoPS rates and mTOR phosphorylation status in the damaged leg were 24% and 19% greater, respectively, postexercise, and 30% and 25% greater, respectively, overnight. Presently, free-living postexercise myoPS rates in the damaged leg of 0.14 ± 0.01%·h^-1^ agree with myoPS rates of 0.13 ± 0.01%·h^-1^ measured in a damaged leg elsewhere using an intravenous stable isotope-labeled amino acid infusion (11). In turn, postexercise myoPS rates in the damaged leg are comparatively greater to myoPS rates measured elsewhere using deuterated water following a bout of traditional, nondamaging, resistance-type exercise (0.08 ± 0.02%·h^-1^) (34). Liberating amino acids from the skeletal muscle reservoir is considered to stimulate protein synthesis and promote wound healing in burn patients (35). It is possible that elevated myoPS rates in the damaged leg are also the result of an increase in endogenous amino acid availability because of elevated muscle protein breakdown. This is consistent with the observation that myoPS rates after eccentric contraction protocols are comparatively greater than those reported after resistance exercise protocols that don’t induce damage (33, 34).

Despite a robust and reproducible stimulation of myoPS and anabolic signaling by ECs, providing additional PPs prebed did not further increase overnight myoPS rates. Protein-polyphenol ingestion significantly increased total daily protein intake compared with PLA (from 1.2–1.8 g·kgBM^-1^·d^-1^; Table 1), an increase associated with potentiated muscle anabolism to resistance exercise training (36). Moreover, PP ingestion provided 20 g of total postexercise protein, which is known to maximally potentiate postexercise myoPS rates (37). Indeed, we have previously shown that daily postexercise PP ingestion attenuates the decline in free-living daily myoPS rates that occurs with daily resistance exercise (16). This would suggest that myoPS rates are refractory to exogenous amino acid availability after ECs, possibly because amino acid requirements are met endogenously via muscle protein breakdown. Alternatively, while we demonstrate that deuterated water can be used to sensitively determine the effect of prior ECs on myoPS rates over just a 3-hour period (Fig. 4A), this approach may not be sensitive to acute changes caused by nutrition, and further investigation using an intravenous stable isotope tracer approach could be required. However, a recent report has also demonstrated that accelerated recovery of muscle function after resistance exercise achieved using a protein nutrition intervention occurred with no further increase in myoPS rates when assessed over a 96-hour period, which would support the current findings (38). Mechanistically, postexercise protein ingestion also likely suppresses muscle protein breakdown (39). Similarly, polyphenols have been shown to attenuate to the inflammatory response to muscle damage (40–44), and inflammation both promotes skeletal muscle protein breakdown and inhibits muscle protein synthesis (19). Considering PP ingestion did not further increase myoPS rates, combined ingestion of polyphenols and protein may synergistically accelerate recovery of muscle function by reducing aberrant muscle protein breakdown following damage. Indeed, the observation that postexercise mTOR protein content was greater in the PLA condition relative to the PP condition could reflect elevated mTOR signaling due to an increased abundance of amino acids derived from protein breakdown (Fig. 5A). It is unlikely that attenuated muscle protein breakdown following PP ingestion would be insulin dependent given that the 25 g of carbohydrate contained within in the PLA drink would also cause insulin secretion.

We targeted a priori 21 genes associated with NF-κB transcription known to be upregulated by ECs (8), a decision corroborated by the repeated identification of enriched pathways associated with NF-κB activation from our functional enrichment analysis. Accordingly, a major novel finding of the present study is that *IL1R1* and *IL1RL1* mRNA expression is downregulated with PP ingestion both postexercise (by 60% and 67%, respectively) and overnight (by 83% and 82%, respectively) (Fig. 7B and 7C, respectively). *IL1R1* and *IL1RL1* can mediate NF-κB activation by the proinflammatory cytokine IL-1β in a TRAF6-dependent signaling cascade (45). Indeed, TRAF6-mediated NF-κB activation was the most enriched pathway overnight. Consistent with this observation, the mRNA expression of *CXCL1*, *ICAM1*, and *CEBPD* (Fig. 7C, 7D, and 7G, respectively) which are downstream of IL-1β–dependent NF-κB activation (46–48) were also downregulated by PP ingestion. These genes are widely reported to be expressed following damaging ECs (5, 7, 8, 49). However, to our knowledge, we are the first to report this novel mechanism whereby suppressing NF-κB transcriptional signaling, likely via the inflammatory IL-1β signaling cascade, may accelerate the recovery of muscle function following damage.

The downregulation in mRNA expression of *CXCL1*, *ICAM1*, and *CEPBD* by protein polyphenol ingestion, in addition to postexercise clustering of *CEPBD* with *CCL2* and macrophage colony-stimulating factor *(CSF1)* (Fig. 8A) suggests PP ingestion may accelerate the recovery of muscle function by reducing the magnitude and/or duration of leukocyte activity mediated by NF-κB signaling. Indeed, leukocyte accumulation in damaged tissue correlates with impaired muscle function following ECs (3). Reactive oxygen species, proteolytic enzymes and cytokines such as IL-1β (50) produced by neutrophils and the inflammatory M1 macrophage population promote tissue degradation (51), exacerbating inflammatory NF-κB signaling in a positive feedback mechanism. Polyphenols scavenge free radicals and suppress reactive oxygen species production (52), which may interrupt this positive feedback loop to attenuate the inflammatory response, as NF-κB signaling is redox-sensitive. Polyphenols derived from pomegranate have also been shown to dose-dependently promote a phenotypic switch towards a proresolution M2 macrophage population in mouse models of ageing and atherosclerosis (53), and M2 macrophages resolve inflammation and localize with regenerating skeletal muscle following damage in humans (54). Protein-polyphenol ingestion may, therefore, accelerate remodeling by suppressing NF-κB signaling to restrain aberrant tissue degradation and inflammation by the M1 macrophage population. Indeed, plateaued recovery of muscle function with PLA ingestion coincides with the time course of peak M1 inflammatory macrophage accumulation (24–48 hours), which would support this notion (55).

NF-κB activation can also induce skeletal muscle breakdown via upregulated mRNA expression of genes of the ubiquitin proteasome system (eg, *MURF*1) (56), which is upregulated by ECs (57). Presently, postexercise *MURF1* mRNA expression was upregulated and clustered with the deubiquitinating protein *USP2*, demonstrating tight regulatory control in the ubiquitin-mediated degradation of protein (Fig. 8A). Indeed, the ubiquitin (*UBB*) gene and protein expression have been shown to increase in response to ECs (58, 59), and presently we observed a 20% increase in postexercise *UBB* expression with PLA ingestion. Interestingly, PP ingestion decreased postexercise *UBB* expression by 102% (interaction, *P* = 0.042, FDR > 5%; Supplementary Table S1(24)), suggesting a reduced ubiquitin demand with PP ingestion, which may represent attenuated breakdown. Interestingly, M2 macrophages have been shown to accelerate the hypertrophy of mouse skeletal muscle following atrophy by decreasing *MURF1* expression and attenuating muscle protein breakdown, more so than potentiating muscle protein synthesis (60). This would support our notion that PP ingestion accelerates remodeling predominantly by attenuating NF-κB signaling to reduce aberrant muscle protein breakdown rather than by increasing myoPS rates. This highlights the need for future work to directly assess muscle protein breakdown after ECs. However, whether PP ingestion directly orchestrates NF-κB signaling to accelerate remodeling, which is plausible considering attenuated NF-κB signaling immediately preceded the recovery of muscle function, or if attenuated NF-κB signaling is simply a reflection of the remodeled state of the muscle brought about by alternative mechanisms requires further investigation. For example, it is feasible that postexercise whey protein ingestion increased myoPS rates in the immediate posteccentric contraction period, which could be crucial in stabilizing myofibrillar integrity and attenuating the requirement for a pronounced inflammatory NF-κB response. To this end, if we calculate myoPS rates between a baseline blood sample taken immediately before deuterated water loading (ie, 11 hours before eccentric contractions) and 24 hours after eccentric contractions using baseline plasma deuterium enrichment as an index of baseline myofibrillar protein-bound [^2^H]alanine enrichment (all participants were deuterium naïve) as has been done previously (34), the increase in myoPS rates between the injured and control leg in PLA (16%) and PP (10%) conditions is similar. While this would support our conclusion that myoPS rates do not underpin the recovery of muscle function after ECs, this calculation relies on a number of assumptions and, therefore, this would require further investigation. Finally, processes associated with, for example, calcium handling, neuromuscular junction integrity, and motor unit recruitment may also be important in governing the recovery of muscle function after ECs, and this would require further investigation (61).

We probed alternative mechanisms comprising the remodeling program with an RT-qPCR gene array coupled with exploratory pathway analysis. We conservatively corrected for false discovery, which yielded only genes demonstrating a time effect. During postexercise and overnight periods, our gene list contained 91% (20/22 genes) of the genes that we have previously shown in a separate study to be upregulated in response to the same eccentric contraction protocol, demonstrating remarkable reproducibility (16). FOXO-mediated transcription (*AKT2*, *MURF1*, and *PPARGC1A*) was enriched exclusively postexercise, consistent with reports elsewhere (62). This may result from weakened insulin signaling, as FOXO-mediated transcription is negatively regulated by insulin and transient insulin resistance has been reported to occur for at least 48 hours in skeletal muscle following a bout of ECs (63). Indeed, impaired Akt activity and a trend for increased Akt protein content has been reported 24 hours following ECs, possibly due to increased inflammatory TNF-α signaling (64), corroborating our observation of upregulated *AKT2* transcription.

In conclusion, targeted postexercise and presleep PP drinks led to a profound increase in the rate of recovery of muscle function, restoring baseline muscle function within 48 hours of maximal damaging ECs. This occurred in the absence of an increase in myoPS rates, but instead we showed for the first time that PP ingestion downregulated mRNA expression of genes indicative of a downregulation in inflammatory IL-1β–mediated NF-κB activation. Downregulated NF-κB signaling preceded the return of baseline muscle function, demonstrating PP ingestion likely accelerates the recovery of muscle function by attenuating aberrant muscle protein degradation, possibly by restraining the proinflammatory M1 macrophage response. Consequently, the time course of macrophage phenotypes in remodeling skeletal muscle, especially in response to nutritional intervention, requires further investigation.

## Data Availability

The datasets generated during and/or analyzed during the current study are not publicly available but are available from the corresponding author on reasonable request.

## Additional Information

***Disclosures:*** This work was part of a PhD studentship grant supported by the University of Exeter and Beachbody LLC. FBS has received payments as a member of the Beachbody LLC scientific advisory board. The results of the present study are presented clearly, honestly, without fabrication, falsification, or inappropriate data manipulation.
